# Preparation of Polymer-Immobilized Polyimide Films Using Hot Pressing and Titania Coatings

**DOI:** 10.1021/acs.langmuir.1c00605

**Published:** 2021-04-01

**Authors:** Mineo Hashizume, Michihisa Hirashima

**Affiliations:** † Department of Industrial Chemistry, Faculty of Engineering, Tokyo University of Science, 12-1 Ichigayafunagawara-machi, Shinjuku-ku, Tokyo 162-0826, Japan; ‡ Graduate School of Chemical Sciences and Technology, Tokyo University of Science, 12-1 Ichigayafunagawara-machi, Shinjuku-ku, Tokyo 162-0826, Japan

## Abstract

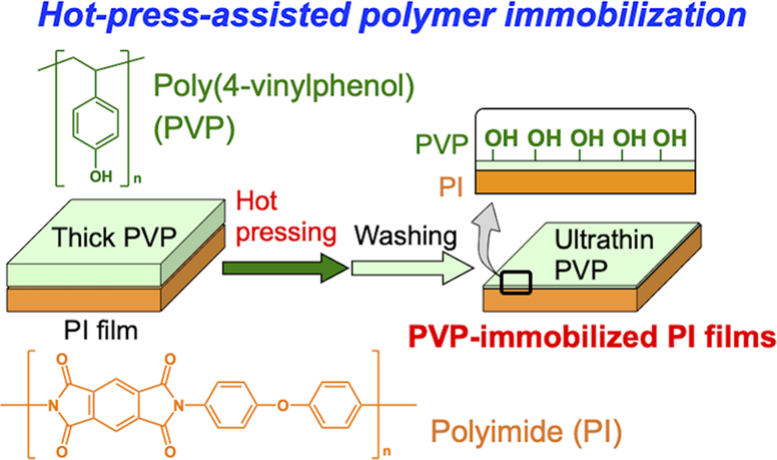

Recent studies have revealed that polymer molecules at film surfaces exhibit unique physical properties compared to those in bulk. On the other hand, such a topic has not been extensively focused for the cases of rigid polymers such as polyimide (PI). This study investigated whether hot pressing could induce the immobilization of other polymers, poly(4-vinylphenol) (PVP), on PI film surfaces. Results supported the immobilization of PVP on the PI film surfaces, and the increase of hot-press temperature resulted in the increase of the immobilization amount of PVP. The mechanism of immobilization is discussed considering the effects of hot pressing on the interactions between PVP and PI at the interfaces of their films. Sol–gel titania coatings were further conducted to the obtained PVP-immobilized PI films. The effect of PVP immobilization on formability and the adhesion of titania layers on the film surfaces were evaluated. These results demonstrate that hot pressing of other polymers is a useful approach for the surface modification of PI films, particularly introducing certain functional groups, and indicate that the polymer immobilization mechanism might be correlated with the surface physical properties of PI films.

## Introduction

To expand the applications of polymer materials, surface modification of polymer substances is an important technology. Various techniques such as plasma treatment,^1−5^ corona discharge,^6,7^ UV/ozone treatment,^8,9^ and so on are often used to generate hydrophilic functional groups on the hydrophobic surface of engineering plastics, including polyolefins, polyesters, and polyimides (PIs). Such surface functionalizations are useful to combine other components, such as metal oxides and their hybrids,^10−13^ bioceramics,^14−16^ and metals,^17−19^ with polymer substrates. Here, the surface modification of polymer substrates is a key point to create organic–inorganic and organic–metal hybrids having robust adhesion interfaces.

The abovementioned techniques are useful and show good performance. They are industrially used to produce polymer materials and their hybrids and composites for practical uses; however, there still remain issues to be improved. For example, some apparatus are big and consume huge energy. Some techniques are not good for the surface modification of nonplanar objects.

Although it is only available for condensation polymers, hydrolysis is an easy technique for the surface modification of polymer substrates. For example, it was demonstrated that the surface-selective hydrolysis of PI films was able to display carboxylate groups on the films, which were utilized for hybrid coatings^20−22^ and adhesion with metal plates.^23^ In these systems, the hydrolysis conditions need to be carefully tuned; excessive hydrolysis causes damage of surface microscopic morphology and decrease of the mechanical properties of the films.

Some new techniques have been utilized for the surface modification of polymer substrates. For example, mussel-inspired polydopamine layers are expected as a ubiquitous coating technique that can achieve good hybrid formation.^24−26^ Material-binding peptides^27,28^ can be utilized as surface modifiers, including those that act as the linkers for hybrid formation for specific polymer substrates.^29^ In these systems, the driving forces of the adhesion of the coating layers or molecules to the polymer substrates are noncovalent interactions. Pristine (as-prepared) polymer substrates are available. In other words, these techniques do not involve the chemical reaction (oxidation, chain scission, and so on) of parent polymers. This study is also categorized in the physical surface modification of polymer substrates.

We have been interested in the fact that the physical properties of the polymer molecules located at the surface of the polymer substrates or in ultrathin films are different from those of polymer molecules in the bulk.^30−34^ For example, Kajiyama, Takahara, and Tanaka et al. have extensively investigated this point. They have demonstrated that the glass transition temperature (*T*_g_) of polystyrene (PS) at the surface of thin films was lower than that in bulk.^35,36^ They have also demonstrated that PS molecular chains at the interfaces of two laminated PS layers are mobile at temperatures below the bulk *T*_g_.^37,38^ Other unique surface properties such as swelling in nonsolvents have also been investigated for other polymers such as poly(methyl methacrylate).^39,40^ This knowledge has inspired us with an idea: even for the case of thin films of “rigid” polymers, the molecules located at the outermost surface might be mobile to some extent at a temperature lower than their bulk *T*_g_. If so, it may be possible to induce entanglement with other polymer molecules at the interface. This is one of the motivations of the present study.

**Figure 1:**
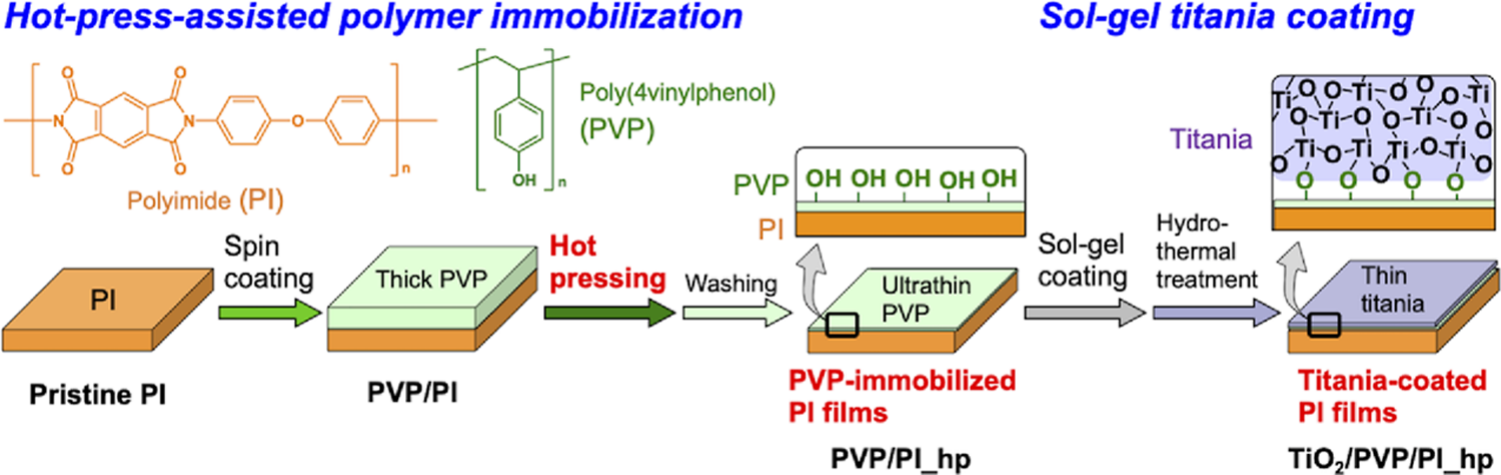
Schematic illustration of this study. Sample labels are also indicated.

PI, a thermally stable super-engineering plastic, can be regarded as a rigid polymer. PI is insoluble in most of the solvents used for the casting of common plastics such as PS. Generally, PI films and substrates are formed by the solution casting of poly(amic acid), an open ring form of PI, followed by heating to form imide rings: direct solution casting and molding of PI are hardly achieved. The tensile moduli of commercial PI films are *ca*. 3 GPa, and such PI films do not show clear *T*_g_ up to 300 °C.^41,42^ In this study, we investigated whether other polymers could be immobilized on PI film surfaces by hot pressing. We chose poly(4-vinylphenol) (PVP) as the immobilizing polymer because it has aromatic groups that may contribute π-π stacking interactions with PI molecular chains. It also has phenolic hydroxy groups that can be reaction sites with titanium alkoxides. Figure 1 shows the schematic illustration of this study. PVP layers were formed on the PI film surfaces and then hot-pressed. Nonimmobilized PVP molecules were extensively washed, and the resulting surfaces were examined by various characterizations. The immobilized PVP molecules on the PI film surfaces could display hydroxy groups to react with titanium alkoxides during the coating of titania layers prepared using sol–gel processes. We demonstrate that hot-press-assisted polymer immobilization is a useful technique for the surface modification of inert polymer substrates, without causing damages to the parent substrates by chemical reactions. This realizes displaying suitable functional groups for additional inorganic coatings thereon. The polymer immobilization mechanism is also discussed.

## Materials and Methods

### Materials

PVP (*M*_w_: 8000), benzene (anhydrous, 99.8%), and quinuclidine were obtained from Sigma-Aldrich Japan Inc. [(*S*)-(−)-4-(*N*,*N*-Dimethylaminosulfonyl)-7-(2-chloroformylpyrrolidin-l-yl)-2,1,3-benzoxadiazole] (DBD-COCl)^43^ was purchased from Tokyo Chemical Industry Co., Ltd. Titanium *n*-butoxide (TNBT) was obtained from Gelest, Inc. Other chemicals were purchased from Nacalai Tesque, Inc. and Kanto Chemical Co., Inc. All chemicals were used as received. PI film (Kapton 100H; Du Pont-Toray Co., Ltd.) was purchased from AS ONE Corp. Distilled water and ultrapure water were prepared and used for the experiments (RFD210TA and RFU414BA, respectively; Advantec Toyo Kaisha Ltd.).

### Preparation of PVP-Immobilized PI Films

PI films were cut into pieces (2.5 × 2.5 cm^2^), and their surfaces were washed in ethanol upon ultrasonication for 30 min using a bath-type sonicator (USM, AS ONE Corp.). The washed PI films were called as “pristine PI”. For PVP thick-layer coating, pristine PI samples were set on a spin coater (1H-D2, MIKASA), and ethanol was spin-coated on their surface (2000 rpm, 30 s) for cleaning. PVP solutions (5, 10 mg mL^–1^ in ethanol, 50 μL) were then spin-coated on the surfaces to obtain PVP-coated PI films (denoted as PVP/PI). Next, the PVP/PI samples were placed on the hot-press stage of the apparatus (AH-2003; AS ONE Corp.). PTFE sheets (Naflon tape with 0.1 mm thickness; AS ONE Corp.) and aluminum foil were inserted between the samples and the stage, for both the top stage and the bottom ones, to prevent contamination. The samples were then pressed at a predetermined temperature (room temperature (r.t.), 120, 140, 160, 180 °C) with the pressure of 10 MPa for 30 min unless otherwise stated. After hot pressing, the samples were removed from the stage and cooled to room temperature. The obtained samples were labeled as “PVP/PI_hp”.

### Fluorescence Labeling of PVP on PI Films

The fluorescence labeling of the phenolic hydroxy groups of PVP immobilized on PI films was conducted according to a previous study.^43^ The benzene (anhydrous) solutions of DBD-COCl (25 mM) and quinuclidine (50 mM) (both were prepared in well-dried vials) were mixed with the ratio of 1:1(v/v) using a vortex mixer, just before use. Pristine PI (as control) and PVP/PI_hp samples (cut into 1 × 1 cm^2^) were then immersed in the mixed solution and incubated at 60 °C (using a water bath) for 15 min. After the reactions, the samples were rinsed in benzene upon ultrasonication for 30 min. The obtained samples were denoted as DBD-PI and DBD-PVP/PI_hp.

### Titania Coating on PVP-Immobilized PI Films

Titania layers were formed on the film surfaces using the sol–gel spin-coating technique.^20,44^ TNBT solutions were prepared using toluene (10 and 100 mM). The PI and PVP/PI_hp samples were set on a spin coater, and ethanol was spin-coated for cleaning. TNBT solutions were then spin-coated on the surfaces (5000 rpm, 120 s) to form amorphous titania layers. The samples were left for several hours to proceed the hydrolysis and polycondensation of the alkoxide layers by air moisture. The resulting samples having amorphous titania (a-TiO_2_) layers were denoted as a-TiO_2_/PI and a-TiO_2_/PVP/PI_hp. To increase the crystallinity of the titania layers, the samples were hydrothermally treated^45,46^ (150 °C, 5 h) using PTFE-lined stainless-steel closed vessels (TAF-SR, Taiatsu Techno Corp.). The treated samples were labeled as TiO_2_/PI and TiO_2_/PVP/PI_hp, respectively. In some cases, much thicker titania layers were formed by repetitive spin coating (10 cycles) on the PVP/PI samples ((a-TiO_2_)_10_/PVP/PI_hp), which were then hydrothermally treated to obtain (TiO_2_)_10_/PVP/PI_hp. For the evaluation of the titania layers of TiO_2_/PVP/PI_hp, UV light (254 nm, 9 W) was irradiated using a handy UV lamp (SLUV-6, AS ONE Corp.) for 20 min. The hydrophilization of the titania layers using this treatment was assessed.

### Characterizations

The water contact angles of the samples were collected using an apparatus (DM-301; Kyowa Interface Science, Co., Ltd.). Sessile drops (0.5 μL) were placed on 10 different areas of each film, and the contact angles (θ/°) were obtained as mean ± standard deviation (SD). Fourier-transform infrared (FT-IR) spectra were collected using a single reflection attenuation total reflection (ATR) method with an apparatus (Nicolet 380; Thermo Fisher Scientific Inc.) combining a Smart Orbit module (Thermo Fisher Scientific Inc.). The elemental compositions of the sample surface region were examined using X-ray photoelectron spectroscopy (XPS, JPS-9010MS; JEOL Ltd.). MgKα radiation (1253.6 eV) was used as the X-ray source. The spectra were obtained at the acceleration voltage of 10 kV, and the take-off angles were set to 15, 30, and 90°. The elemental compositions of the samples were calculated using the peaks of C (1 s), O (1 s), N (1 s), and Ti (2p_3/2_) that were obtained using the narrow mode. The correction of binding energy was conducted using the peak of the N-C species (400.5 eV) originating from PI in the N region of the narrow spectrum. The data were analyzed using the equipped software (SpecSurf; JEOL Ltd.). (In some cases, the peak deconvolutions of the C 1s regions were examined.) The peaks were assigned according to the database in the software and the literature.^47,48^ The morphology of the sample surfaces was evaluated using scanning electron microscopy (SEM, S-5000; Hitachi Ltd. or JSM-7001; JEOL Ltd.) with the acceleration voltages of 2–10 kV. The specimens were coated with Pt–Pd using an ion-sputtering device (E-1030; Hitachi Ltd.). For some cases, energy-dispersive X-ray spectroscopy (EDX) was simultaneously conducted with SEM observations (Sigma, Kevex).

The fluorescence spectra of DBD-PI and DBD-PVP/PI hp were collected using an apparatus (FP-8500, Jasco, Ltd.). The samples were attached on the support substrates that were placed in the cuvette holder of the apparatus with 45° direction toward the incident light source.

### Evaluation of the Adhesion Strength of Titania Layers

For the evaluation of the adhesion strength between titania layers and PVP-immobilized PI films, thicker titania layers were formed on PVP/PI_hp. TNBT solutions (100 mM) (in ethanol, 50 μL) were placed on the pristine PI and PVP/PI_hp surfaces, maintained for 40 s, and then spin-coated (5000 rpm, 120 s). The obtained samples, a-TiO_2_/PI and a-TiO_2_/PVP/PI_hp, were hydrothermally treated, as described above, to obtain TiO_2_/PI and TiO_2_/PVP/PI_hp, respectively.

The obtained samples were subjected to tape tests. The strips of Scotch mending tape (810–3-18; 3M) were adhered to the surfaces (titania face) of the samples, and their surfaces were rubbed using an eraser and then peeled off manually. This adhesion–peel off process was repeated two times for each sample. After the treatment, the sample surfaces were evaluated by SEM.

The 90° peel tests were also examined using a universal tester (Autograph AGS-J; Shimadzu Corp.) with a 50 N load cell. One edge of the long strips of the tapes was adhered to the surfaces (titania face) of the samples that are fixed on the apparatus using the support substrates, and the tape surfaces were rubbed using an eraser. The tapes were then peeled off by pulling up their free edges in the upward direction with a speed of 10 mm min^–1^. The stress–stain curves were collected and analyzed using a TRPEZIUMX software. The obtained numerical data such as the maximum tensile strength were expressed as mean ± S.D.

## Results and Discussion

### Preparation of PVP-Immobilized PI Films

**Figure 2:**
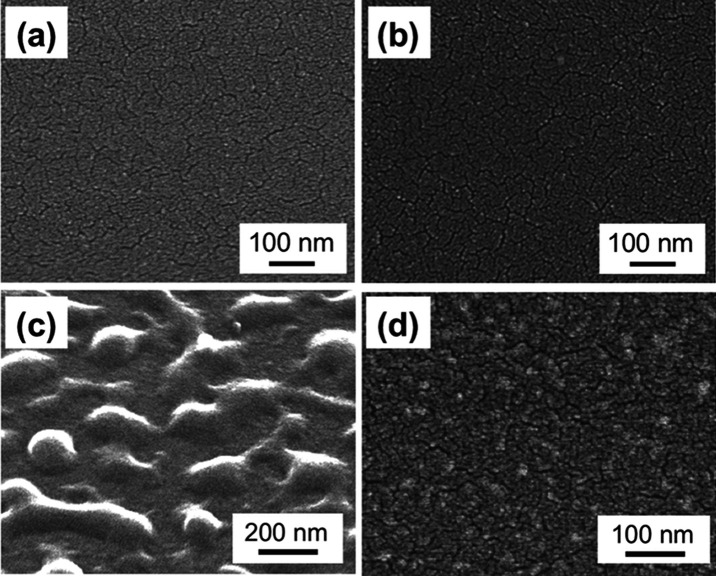
SEM images of the surfaces of pristine PI (a), hot-pressed (180 °C, 10 MPa, 30 min) PI (b), PVP-coated PI (PVP/PI c), and PVP-immobilized (hot press condition: 180 °C, 40 MPa, 30 min) PI (PVP/PI_hp, d) films.

(a) Effect of hot-press temperature. As shown in Figure 1, in this study, we investigated whether hot pressing could induce the entanglement of polymer chains at the interface between the PVP layers and PI films. PVP was readily spin-coated on the pristine PI films, which resulted in the formation of thick PVP layers on the PI film surfaces (PVP/PI). The samples were then hot-pressed, followed by extensive washing to remove excess PVP molecules that were not immobilized on the PI film surfaces. The hot-pressed samples (PVP/PI_hp) were evaluated by SEM. Hot-pressing itself did not affect the surface microstructures of the PI films (Figure 2a,b), in addition to their atomic compositions (Figure S1). Therefore, we concluded that the so-called “anchor effect”, caused by surface roughness, did not contribute the PVP immobilization of PI film surfaces. The surface of PVP/PI (Figure 2c), corresponding to the sample before hot pressing (Figure 1), showed that the PVP layers spin-coated on PI film surfaces had a rough morphology. This might come from the fact that the spin-coating condition was not optimized for the preparation of microscopically flat thin PVP films. Because we focused on the interactions between PVP and the PI molecules at the interfaces of their layers, we have not measured the thickness of the spin-coated PVP layers. In Figure 2c, the bottom of the concaves looked different from the PI film surfaces, indicating that a sufficient thickness of PVP layers for the immobilization of PVP molecules was formed on the PI film surfaces. On the other hand, the surfaces of the hot-pressed samples (180 °C, 40 MPa), PVP/PI_hp (Figure 2d), were flat and totally different from those of PVP/PI (Figure 2c). The results supported that excess PVP layers were removed by the washing process. The microscopic surface morphology of the PVP/PI_hp samples looked similar to that of the pristine PI films (Figure 2a), but the differences in contrast were observed in the surfaces, which might indicate the immobilization of PVP molecules on the film surfaces. It was found that 10 MPa was enough to immobilize PVP; therefore, the following investigations were mainly conducted for samples prepared using 10 MPa of hot pressing. The water contact angle (θ) of PVP/PI_hp was 58.4 ± 3.3°. It was significantly smaller than that of pristine PI (74.6 ± 1.0°). Referring to the SEM results, it was supported that this hydrophilization was not due to surface roughening but came from the immobilization of PVP on the PI film surfaces by hot pressing.

**Figure 3:**
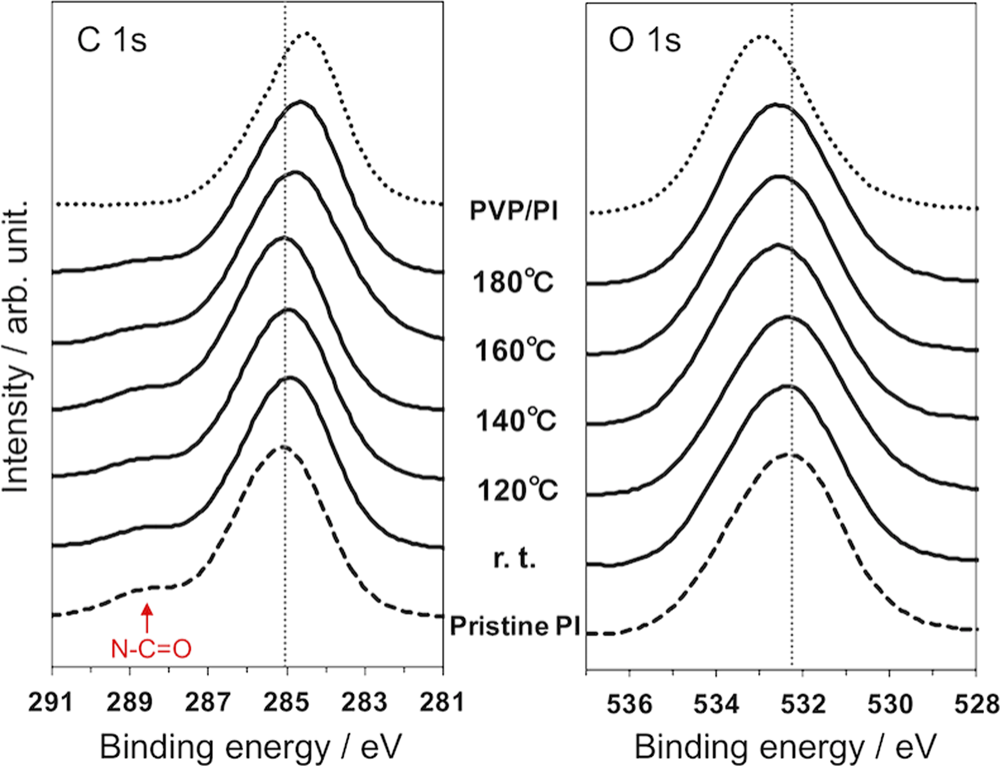
XPS spectra of the C 1 s region (left) and O 1 s region (right) of pristine PI, PVP/PI, and PVP/PI_hp with different hot-press temperatures. Vertical dotted lines indicate the positions of the peak tops for pristine PI. Samples were prepared using 5 mg mL^–1^ of PVP solutions. Take-off angle: 15°.

It was difficult to confirm the immobilization of PVP molecules on the PI film surfaces for the PVP/PI_hp samples by FT-IR spectroscopy (data not shown) because the expected amounts of PVP were small. Figure 3 shows the XPS spectra of the PVP/PI_hp samples prepared using different hot-press temperatures (PVP concentration of the coating solution was 5 mg mL^–1^). Because PVP does not contain characteristic hetero atoms, the shape of the spectra of C and O was evaluated. As for the C 1 s regions, in addition to the main peak originating from aromatic C, the small peak corresponding to the carbon of N–C=O was found for pristine PI films.^47,48^ The intensities of this peak decreased with the increase of the hot-press temperature, accompanying with the shift of the peak top of the main peak. The main peak became close to that of PVP/PI, whose surface was covered by thick layers of PVP that contains aliphatic C. These results indicated that the increase of the hot-press temperature increased the amount of PVP on the PI film surfaces. Unfortunately, an accurate curve fitting of the spectra to distinguish aromatic and aliphatic carbon atoms was difficult because of a large overlap of these peaks. The stack spectra of the O 1 s regions for the corresponding samples also demonstrated the shift of the peak tops toward that of PVP/PI with the increase of the hot-press temperature. The peak of PVP/PI was mainly contributed by the phenolic hydroxy groups of PVP on the PI films. These results also supported the increase of the amount of PVP on the PI films with an increasing hot-press temperature. The immobilization of PVP on the PI film surfaces should change the surface atomic composition. Table 1 shows the summary of the atomic compositions of the samples calculated using the XPS spectra. Compared to pristine PI, the carbon composition of PVP/PI_hp increased while the nitrogen composition decreased. The increase of carbon composition was intensified for PVP/PI_hp hot-pressed at 180 °C. This also indicated PVP immobilization, in addition to the clear peak shift in the spectra (Figure 3). When pristine PI films were directly hot-pressed at 180 °C, 10 MPa for 30 min, the atomic composition of the resulting sample was C: 77.8%, N: 4.7%, and O: 17.5%. Hot pressing itself did not change the chemical composition of the PI film surfaces significantly (Figure S1), same as the microscopic morphology, as supported by the SEM observations (Figure 2). On the other hand, although PVP/PI_hp (r.t.) showed an increase in the carbon composition, the peak shift was not significant. Also, the oxygen composition did not show a clear tendency. This might indicate the limit of the detailed evaluation by XPS; even so, the present results supported PVP immobilization on the PI film surfaces by hot pressing.

**Table 1:** Atomic Compositions of Pristine PI and PVP/PI_hp Samples^a^^,^^b^

sample	C/%	N/%	O/%
pristine PI	76.5	5.3	18.2
PVP/PI_hp (r.t.)	79.7	4.0	16.3
PVP/PI_hp (120 °C)	78.7	3.5	17.8
PVP/PI_hp (140 °C)	77.4	3.7	18.9
PVP/PI_hp (160 °C)	78.5	2.3	19.2
PVP/PI_hp (180 °C)	81.6	2.8	15.7
PI (theoretical value)	75.9	6.9	17.2
PVP (theoretical value)	88.9		11.1

a Atomic compositions were calculated using C, O, and N (C + N + O = 100%).

b XPS spectra used for calculations were obtained using the take-off angle of 15°.

**Figure 4:**
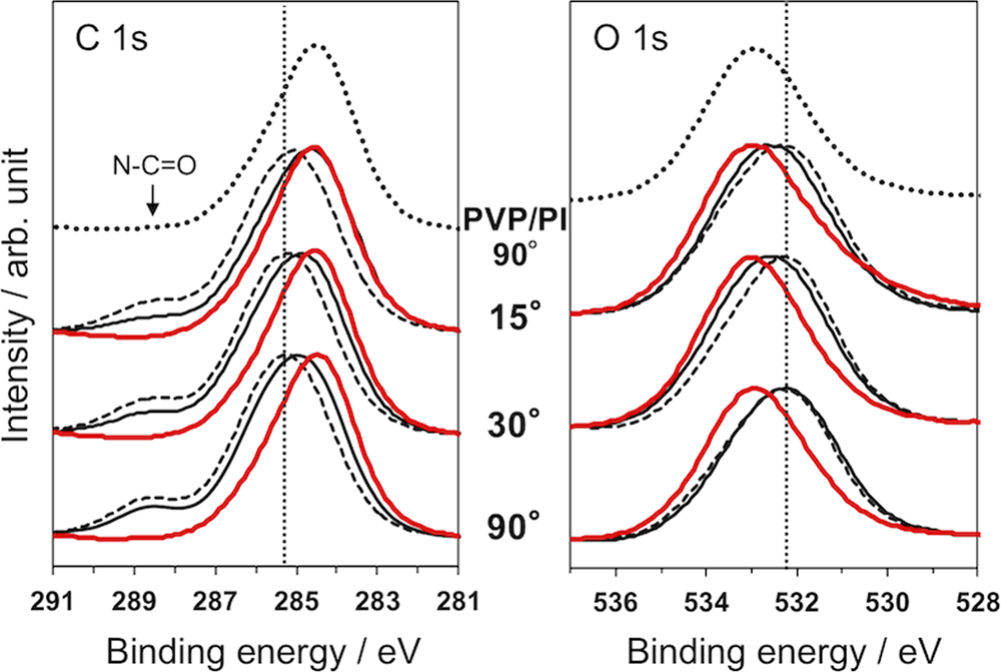
XPS spectra of the C 1 s region (left) and the O 1 s region (right) of pristine PI (dashed lines) and PVP/PI_hp samples using different take-off angles. PVP/PI_hp samples were prepared using 5 mg mL^–1^ (black solid line) and 10 mg mL^–1^ (red solid line) of PVP solutions. Results for PVP/PI (PVP: 5 mg mL^–1^, take-off angle: 90°) are also indicated. Vertical dotted lines indicate the positions of the peak tops for pristine PI. Hot pressing: 180 °C, 10 MPa, 30 min.

(b) Effect of PVP concentration. When the concentration of the PVP solutions used for spin coating was increased from 5 mg mL^–1^ (black solid line) to 10 mg mL^–1^ (red solid line), larger peaks shifts were observed in the XPS spectra (Figure 4). The results indicated that the increase of PVP solution concentrations resulted in the immobilization of a larger amount of PVP on the PI films surfaces by hot pressing. Although we thought that a sufficient thickness of PVP layers was formed on the PI film surfaces when using 5 mg mL^–1^ PVP solutions (see the above description about Figure 2c), the density of PVP on the PI film surfaces might be less compared to the case of using 10 mg mL^–1^ solutions. The atomic compositions obtained from these spectra were summarized as a function of the take-off angle (Figure S2). The graph revealed that the increase of PVP concentration increased the carbon composition and decreased the nitrogen and oxygen compositions, indicating the increase of PVP immobilization. These results also give information about differences in the atomic composition in-depth direction. Generally, detection using the lower take-off angle gives information closer to the surface. For 5 mg mL^–1^ of PVP, the difference of atomic composition between 15 and 30° was remarkable, whereas that between 30 and 90° was moderate. For 15°, the carbon composition increased more and the nitrogen and oxygen compositions decreased more. This could be interpreted as that PVP existed close to the outermost surfaces of the PI films, up to a few nanometer regions. On the other hand, for 10 mg mL^–1^ of PVP, the atomic compositions of 15, 30, and 90° were similar, and they were larger (for C) and lower (for N and O) than those of 5 mg mL^–1^ of PVP. These results indicated that more PVP molecules were immobilized on the surface region of the films compared to the case of 5 mg mL^–1^ of PVP. There are two possibilities for this: PVP molecules were more deeply penetrated into the PI films or PVP layers were formed on the PI film surfaces. From these results, it was concluded that the optimized condition was that using 10 mg mL^–1^ of PVP solutions and 180 °C, 10 MPa, 30 min of hot pressing. The PVP/PI_hp samples used for the following experiments were obtained using this condition.

(c) Fluorescence labeling of PVP on PI films. DBD-Cl is used for the fluorescence labeling of nucleophilic functionalities including phenolic hydroxy groups.^43^Figure S3 shows the fluorescence spectra of pristine PI and PVP/PI_hp treated with DBD-Cl. The spectrum of DBD-PVP/PI_hp supported the coupling of DBD groups with the phenolic hydroxy groups of PVP immobilized on the PI film surfaces. Unexpectedly, DBD-PI also showed a moderate fluorescence intensity. This might come from the nonspecific adsorption of DBD-Cl or its hydrolyzed products. The difference in the maximum emission wavelength indicated the difference in the chemical state of the DBD groups, and the blue-shifted emission by coupling with the phenolic hydroxy groups was in good agreement with the literature.^43^

### Mechanism of PVP Immobilization

The results shown above supported that PVP was immobilized on the PI film surfaces by hot pressing. Because PVP was chemically stable up to 220 °C, as evaluated by FT-IR spectroscopy (data not shown), the chemical bond formation between PVP and PI molecules was not the main factor for PVP immobilization. The immobilization was probably based on noncovalent interactions. Here, π-π stacking interactions were one considerable driving force. Two types of π-π stacking interactions might be possible between PVP and PI in the present case. One is that the phenyl rings of PVP face the PI film surfaces and stack the phenyl rings of PI that exist parallel to the PI film surfaces (face-to-face-type stacking interaction). Such an interaction mode was possible without the interpenetration of PVP and PI at the interfaces of their films. Interactions between the π-rich 2D surfaces and polymers having aromatic groups were reported for graphene-type materials and poly(sodium 4-styrene sulfonate) (PSS) systems.^49,50^ On the other hand, if interpenetration or entanglement of PVP and PI molecular chains occurs at their film interfaces during hot pressing, the π-π stacking interactions between the phenyl rings of PVP and PI can be achieved at any directions inside the PVP/PI mixing layers. This is the second type of π-π stacking interactions. During the hot-press processes, pressing of the samples enhanced the physical contact between PVP and PI at their film surfaces, which increased the probability of the occurrence of π-π stackings. As for heating, it should be noted that the increase of hot-press temperature resulted in the increase of the PVP immobilization amount. Because the *T*_g_ of PVP was 135–180 °C,^51^ a higher hot-press temperature (close to 180 °C) could affect the physical properties of the PVP layers on the PI film surfaces to increase the physical contact to PI, following face-to-face-type π-π stacking. This is one possible and reasonable explanation. As for the heating effect on PI, PI (Kapton) does not show a clear *T*_g_ up to 300 °C,^41,42^ and there is no report regarding the surface *T*_g_ of the PI films. It appeared to be difficult to affect the physical properties of the PI films by heating up to 180 °C. On the other hand, the surface dynamic property of the PI films is described in the field of the development of liquid crystal (LC) displays. It is known that the rubbing of the PI film surfaces induces the alignment of LCs thereon, and reports propose that this is because of the alignment of PI molecular chains by rubbing.^52−54^ It should be noted that the rubbings were conducted at room temperature. These facts indicate the dynamic nature of PI molecular chains at the outermost surface of the PI film, which might accept interpenetration with other polymers thereon. At this moment, a direct proof of π-π stacking has not been obtained, but further investigations will clarify the structural requirements of the immobilizing polymers.

### Titania Coating on the PVP-Immobilized PI Films

**Figure 5:**
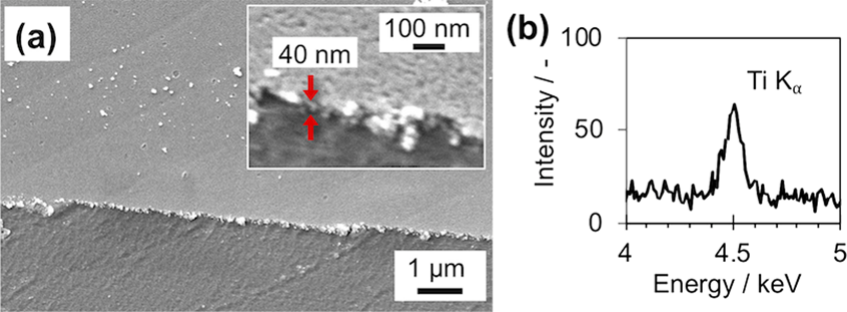
SEM images (a) and EDX spectrum (b) of the surface of TiO_2_/PVP/PI_hp prepared using 100 mM TNBT solutions. In (a), the inset is an image of higher magnification, and arrows are shown to indicate the apparent thickness of the titania layer.

If PVP molecules are immobilized on the PI film surfaces, it is expected that they present hydroxy groups on the surfaces that can contribute to adhesion between the inorganic layers, subsequently formed using various solution processes, and PI films. For the case of sol–gel titania coating, the hydroxy groups of PVP on PI films can react with TNBT to form covalent bonds. The sol–gel reaction of TNBT resulted in the formation of titania layers covalently bonded to the PVP-immobilized PI films (Figure 1). To examine this point, sol–gel titania coatings on the PVP/PI_hp samples were evaluated. First, the formability of titania layers on PVP/PI_hp was checked. Figure 5 shows the SEM images and the EDX spectrum of the surface of titania-coated, PVP-immobilized PI films after hydrothermal treatments (TiO_2_/PVP/PI_hp) prepared using 100 mM TNBT solutions. The results supported the formation of homogeneous titania layers on PVP/PI_hp. When looking at the edge of the layers that sometimes happen to be formed by sol–gel spin coatings at higher magnifications, the layers consisted of granules of titania and had thicknesses of about several tens of nanometers (Figure 5a, inset). The effect of the TNBT concentration of spin-coating solutions, spin coating times, and hydrothermal treatment on the formability of the morphology of titania layers were then examined (Figure S4). For all the examined samples, titania layers were formed on the PVP-immobilized PI film surfaces. The microscopic morphology and the thickness of the titania layers were different depending on the preparation conditions. Although it is hard to say that there was a linear correlation between the spin-coating times or TNBT concentration and the thickness of the resulting titania layers, these results showed that homogeneous titania layers were able to be formed on the PVP/PI_hp sample surfaces.

The formation of titania layers was also checked by UV-light irradiation. The water contact angles of a-TiO_2_/PVP/PI_hp and TiO_2_/PVP/PI_hp, prepared using 100 mM TNBT solutions, were 69.3 ± 1.4° and 68.5 ± 2.4°, respectively. After UV-light irradiation, the angles changed to 20.9 ± 5.2° and 13.9 ± 4.2°, respectively. The results reflected the hydrophilization of titania by UV irradiation, an intrinsic feature of titania.^55^

### Adhesion Strength of the Titania Layers

**Figure 6:**
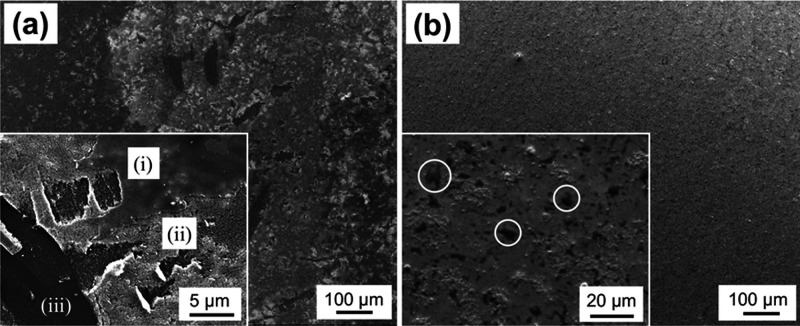
SEM images of the surfaces of TiO_2_/PI (a) and TiO_2_/PVP/PI_hp (b) after tape peel tests. Insets are magnified images. In (a), (i), (ii), and (iii) indicate the regions of the original titania surface (i), interpeeling of titania (ii), and the whole detachment of titania (iii), respectively. In (b), the white circles indicate some of the traces of tapes.

To evaluate the effect of PVP immobilization on adhesion between the titania layers and PVP-immobilized PI films, tape peel tests were conducted for the TiO_2_/PVP/PI_hp samples, in addition to the TiO_2_/PI samples as a reference. Titania layers could be formed on pristine PI films. To conduct the tape test, thick titania layers were formed by changing the coating conditions (see the Experimental Section). After peeling the adhered tapes, the surfaces of TiO_2_/PI and TiO_2_/PVP/PI_hp were observed by SEM (Figure 6). Some regions of titania layers remained (region (i) of the inset of Figure 6a), and the interlayer peelings of titania were observed in other regions of the titania layers (region (ii)). In addition, whole detachments of the titania layers were observed in part (region (iii)). The results indicated that the sol–gel-coated titania layers formed on the pristine PI film surfaces were not so robust, probably because the driving force of adhesion was mainly physisorption. Particularly, the appearance of region (iii) supported the weakness of the titania–PI interfaces. On the other hand, homogeneous surfaces were observed for TiO_2_/PVP/PI_hp even after tape peeling (Figure 6b). In the magnified images, dark objects probably correspond to that traces of tapes were found on the titania layers (Figure 6b, inset). The results supported the strong adhesion of the titania layers with the PVP/PI_hp surfaces. The results of 90° peel tests indicated that adhesion forces between the tape and the hydrothermally treated titania layers were stronger than those between the tape and amorphous titania (Figure S5). The constant regions of the adhesion strength were similar between TiO_2_/PI and TiO_2_/PVP/PI_hp, but downward spikes that probably correspond to the detachment of whole titania layers or the interpeeling of titania were found in the profile for TiO_2_/PI (Figure S5a). This was in good agreement with the SEM observations (Figure 6a). These results indicated the linker effect of PVP immobilized on the PI films, which could be explained as follows: TNBT molecules at the film surfaces reacted with the phenolic hydroxy groups of PVP molecules to form Ti–O–phenyl bonding and developed Ti–O–Ti networks during the sol–gel process, which realized the whole immobilization of the titania layers to PVP that physically immobilized on the PI film surfaces. Therefore, adhesion between the titania layers and PVP-immobilized PI films was achieved, as illustrated in Figure 1.

## Conclusions

This study demonstrates that PVP molecules could be physically immobilized on the PI film surfaces by hot pressing. Also, the immobilized PVP could act as the reaction sites for sol–gel-coated titania layers on the films. We want to emphasize that this process is different from the so-called melt mixing. We are currently conducting the immobilization of other polymers having effective functional groups for additional coating of inorganic components such as bioceramics on PI films using this approach. Such an approach can contribute to the diversification of surface modification techniques for chemically inert, heat-resistant polymer materials, including super-engineering plastics.

## Supporting Information Available

Atomic composition of pristine and hot-pressed PI films obtained from XPS spectra using different take-off angles, atomic compositions of pristine PI and PVP/PI_hp samples obtained from XPS spectra using different take-off angles, fluorescence spectra of pristine PI, DBD-PI, and DBD-PVP/PI_hp, SEM images and corresponding EDX spectra of the surfaces of titania-coated PVP/PI_hp films, and examples of the profiles of 90° peel tests (PDF)
