# Supplementary material for: Preparation of Polymer-Immobilized Polyimide Films Using Hot Pressing and Titania Coatings
Source: Langmuir. 2021 Apr 1;37(14):4403–10. doi: 10.1021/acs.langmuir.1c00605 (PMC8154877; doi:10.1021/acs.langmuir.1c00605)
Supplement: Supplementary file 1 — la1c00605_si_001.pdf [file la1c00605_si_001.pdf]

Preparation of Polymer-Immobilized Polyimide Films Utilizing Hot Pressing and Titania Coatings

Mineo Hashizume[†,‡,*] and Michihisa Hirashima[‡]

[†] *Department of Industrial Chemistry, Faculty of Engineering, Tokyo University of Science, 12-1 Ichigayafunagawara-machi, Shinjuku-ku, Tokyo 162-0826, Japan*
[‡] *Graduate School of Chemical Sciences and Technology, Tokyo University of Science, 12-1 Ichigayafunagawara-machi, Shinjuku-ku, Tokyo 162-0826, Japan*

Supporting Information

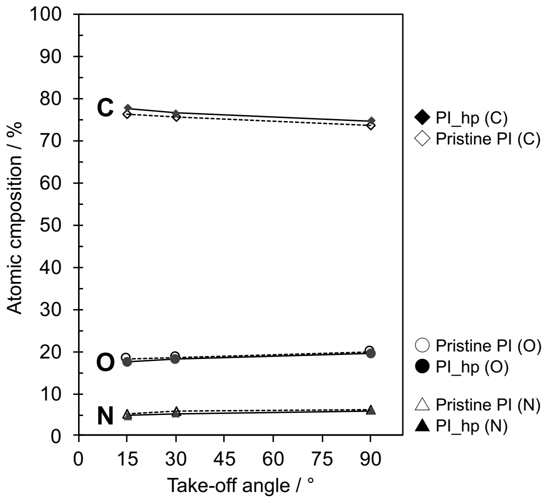

Figure S1. Atomic composition of pristine and hot pressed (PI_hp, 180 °C) PI films obtained from XPS spectra using different take-off angles.

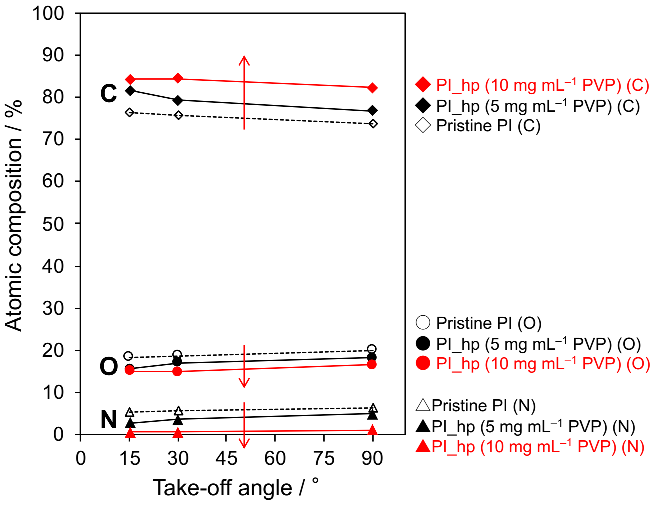

Figure S2. Atomic compositions of pristine PI and PVP/PI_hp samples prepared using 5 mg mL^{-1} and 10 mg mL^{-1} PVP solutions obtained from XPS spectra using different take-off angles.

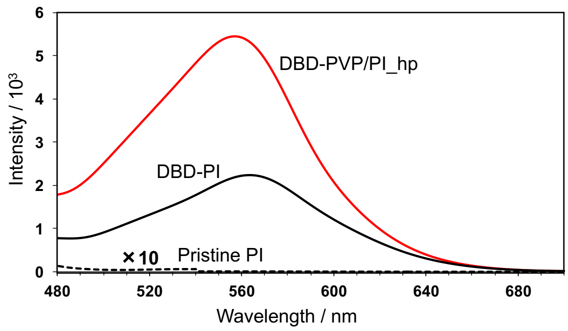

Figure S3. Fluorescence spectra of pristine PI (×10, dashed line), DBD-PI (black solid line), and DBD-PVP/PI_hp (red solid line). Excitation wavelength: 440 nm.

Titania Coating on PVP-Immobilized PI Films Under Various Conditions. Effect of TNBT concentration of spin coating solutions, spin coating times and hydrothermal treatment on formability of morphology of the titania layers were then examined (Figure S4). The procedures were described in the Experimental Section of the main text. When using 10 mM TNBT solutions, SEM observations indicated that one cycle of spin coating of the solution resulted in formation of rough surfaces consisted of amorphous titania (a-TiO_2/PVP/PI_hp (10 mM), Figure S4a). After hydrothermal treatment, granular morphologies were pronounced (TiO_2/PVP/PI_hp (10 mM), Figure S4b). It was hard to judge about the thickness of the thin titania layers. For the case of 10 times repetitive spin coating, the titania layers became thicker and were observable ((a-TiO_2)$_{10}$/PVP/PI_hp (10 mM), Figure S4d). It was clearly shown that the thin titania layers were consisted of granules after hydrothermal treatments ((TiO_2)$_{10}$/PVP/PI_hp (10 mM), Figure S4e). Increase of the titania component on the film surfaces by increasing of spin coating times also supported by EDX spectra of these samples (Figures S4c and S4f). Increasing the concentration of TNBT solutions to 100 mM, one time of spin coating could form homogeneous amorphous titania layers that became granule-assembled structures by hydrothermal treatments (a-TiO_2/PVP/PI_hp (100 mM) and TiO_2/PVP/PI_hp (100 mM), Figures S4g and S4j). The latter is the same condition as to that shown in Figure 5. For 10 times of spin coating of 100 mM solutions, thicker titania layers were formed, as expected ((a-TiO_2)$_{10}$/PVP/PI_hp (100 mM) and (TiO_2)$_{10}$/PVP/PI_hp (100 mM), Figures S4h and S4k). The EDX results were well corresponded (Figures S4i and S4l). It was hard to say that there was a linear correlation between spin coating times or TNBT concentration and thickness of the resulting titania layers. Experimental variations were included in the obtained thicknesses. Even so, these results showed that homogeneous titania layers were able to formed on PVP/PI_hp samples' surfaces.

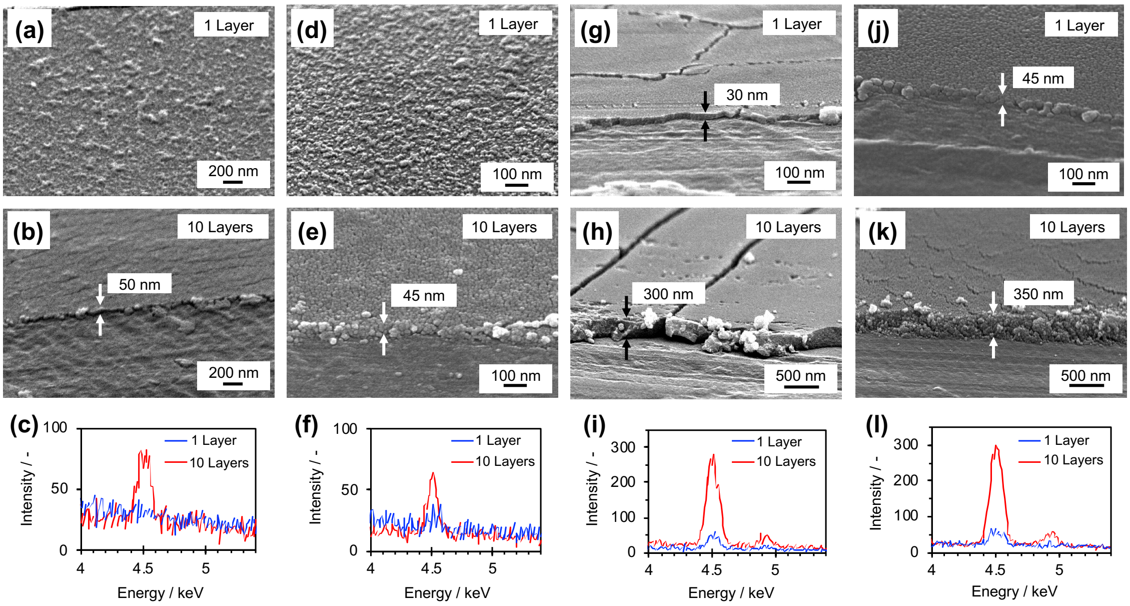

Figure S4. SEM images (a, b, d, e, g, h, j, k) and corresponding EDX spectra (c, f, i, l) of the surfaces of titania-coated PVP/PI_hp films. Samples were prepared using 10 mM (a-f) and 100 mM (g-l) TNBT solutions. As-prepared (a-c and g-i) and hydrothermally-treated (d-f and g-l) samples are shown. Spin coatings were conducted one time (a, d, g, j) and 10 times (b, e, h, k).

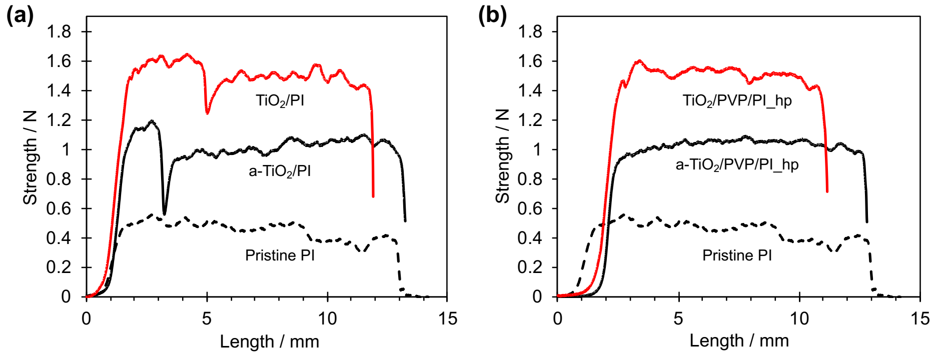

Figure S5. Examples of profiles of 90° peel tests. (a) a-TiO_2/PI_hp and TiO_2/PI_hp and (b) a-TiO_2/PVP/PI_hp and TiO_2/PVP/PI_hp. Results for pristine PI are shown as reference. In (a), spikes indicate detachment of titania layers.
